# An Integrated Model of Environmental Effects on Growth, Carbohydrate Balance, and Mortality of *Pinus ponderosa* Forests in the Southern Rocky Mountains

**DOI:** 10.1371/journal.pone.0080286

**Published:** 2013-11-25

**Authors:** Christina L. Tague, Nathan G. McDowell, Craig D. Allen

**Affiliations:** 1 Bren School of Environmental Science and Management, University of California Santa Barbara, Santa Barbara, California, United States of America; 2 Earth and Environmental Sciences Division, Los Alamos National Laboratory, Los Alamos, New Mexico, United States of America; 3 United States Geological Survey, Fort Collins Science Center, Jemez Mountains Field Station, Los Alamos, New Mexico, United States of America; DOE Pacific Northwest National Laboratory, United States of America

## Abstract

Climate-induced tree mortality is an increasing concern for forest managers around the world. We used a coupled hydrologic and ecosystem carbon cycling model to assess temperature and precipitation impacts on productivity and survival of ponderosa pine (*Pinus ponderosa*). Model predictions were evaluated using observations of productivity and survival for three ponderosa pine stands located across an 800 m elevation gradient in the southern Rocky Mountains, USA, during a 10-year period that ended in a severe drought and extensive tree mortality at the lowest elevation site. We demonstrate the utility of a relatively simple representation of declines in non-structural carbohydrate (NSC) as an approach for estimating patterns of ponderosa pine vulnerability to drought and the likelihood of survival along an elevation gradient. We assess the sensitivity of simulated net primary production, NSC storage dynamics, and mortality to site climate and soil characteristics as well as uncertainty in the allocation of carbon to the NSC pool. For a fairly wide set of assumptions, the model estimates captured elevational gradients and temporal patterns in growth and biomass. Model results that best predict mortality risk also yield productivity, leaf area, and biomass estimates that are qualitatively consistent with observations across the sites. Using this constrained set of parameters, we found that productivity and likelihood of survival were equally dependent on elevation-driven variation in temperature and precipitation. Our results demonstrate the potential for a coupled hydrology-ecosystem carbon cycling model that includes a simple model of NSC dynamics to predict drought-related mortality. Given that increases in temperature and in the frequency and severity of drought are predicted for a broad range of ponderosa pine and other western North America conifer forest habitats, the model potentially has broad utility for assessing ecosystem vulnerabilities.

## Introduction

Drought-related forest mortality is a widespread phenomenon and is expected to increase with continued climate warming [1], [2], [3]. Drought is also an important driver of forest productivity and longer-term patterns of growth and forest structure and composition [4]. Models provide tools for investigating productivity and mortality risk under climate change scenarios and are a key source of information for forest management planning and impact assessment [5], [6], [7]. Modeling approaches range from fully mechanistic physiological representations to envelope models that use historic climate-productivity relationships to estimate future responses. While fully mechanistic approaches are better able to disentangle the impact of multiple interacting processes, intermediate complexity carbon cycling models are valuable for estimating watershed to regional scale net primary production (NPP) and have been validated by comparisons with flux-tower and remote-sensing estimates of NPP [8], [9], [10]. Historically, however, forest carbon cycling models do not explicitly represent the physiological mechanisms involved in forest mortality [11].

Recent research has focused on mechanisms that underlie drought-related tree mortality and has identified both endogenous factors such as carbon starvation and hydraulic failure, and exogenous factors such as attack by insect and disease, that lead to death [12], [13], [11]. Identifying the specific mechanisms by which these factors individually or in combination lead to tree death remains an active area of research. Earlier work positioned hydraulic failure and carbon starvation as distinct mortality mechanisms, but more recent investigations suggest mortality may occur through interactions between these and other mechanisms [11], [14]. There is mounting evidence that death can occur prior to complete depletion of non-structural carbohydrate (NSC) reserves, suggesting that accurately modeling within plant transport of NSC may be necessary to predict mortality related to a depletion of energy reserves. Explicit representation of the mortality mechanisms is likely to require representing a combination of drought effects on NSC depletion, within plant transport of NSC, controls on the use of NSC reserves (or sink effects), and their interactions [15], [13], [14]. While models of within plant hydraulics and transport exist [16], the complexity of these models limits their applicability at watershed scales where forest behavior also depends on forest structure and composition patterns, climate gradients, and soil water holding and drainage characteristics.

Coupled models of ecosystem carbon cycling and hydrology offer intermediate complexity in representing plant physiology as well as plot to watershed scale controls on moisture and energy exchange. The challenge is to adapt these models to account for mortality risk without full representation of within plant mortality mechanisms until more mature (and efficiently parameterized) detailed mechanistic models are available [11]. We tested the addition of a simple two-parameter model of NSC storage dynamics to an existing coupled carbon cycling and hydrology model, the Regional Hydro Ecological Simulation System (RHESSys). While NPP declines with water stress, the relationship between daily or seasonal NPP and mortality also depends on energy reserves that can be used for maintenance, repair and defense. Several carbon cycling models include a dynamic NSC pool, but utilize all carbohydrates each day [17] or by the end of each year [18] (see [11] for a review).

In this paper, we explore the seasonal and inter-annual dynamics of the NSC pool, its relationship with climate and water availability, and potential linkages with NPP and mortality. We then use RHESSys with the inclusion of the NSC pool dynamics (RHESSys-NSC) to estimate the likelihood of mortality, and compare results with an observed mortality event at the lower ecotone of a ponderosa pine (*Pinus ponderosa*) distributional gradient [19], [20]. Simulating the tree and stand level NSC pool is only a proxy for more complex mortality mechanisms such as insect attack or hydraulic failure, but photosynthetic and NSC dynamics are strongly associated with each of these processes and with mortality [21], [22], [23], [24]. We use ten years of stem growth data from this site and two higher ponderosa pine sites across an 800 m elevational gradient to evaluate model estimates of productivity, and then use sensitivity analyses to explore linkages between NSC parameters, the likelihood of mortality, and their interactions with site characteristics across the elevational gradient.

## Methods

### Overview

Our approach was to first develop a sub-model of NSC pool dynamics and link it to RHESSys model simulations of photosynthesis and carbon allocation, test the model against observational data of growth and mortality, and then conduct a sensitivity analysis to determine optimal parameterization and the influence of climate and site characteristics on productivity and survival. To test if the modeled carbon cycle was reasonable, we compared predictions of inter-annual variation in NPP with observations of growth as basal area increment (BAI) for each of the three sites through time. We also compare model estimates of cross-site difference in biomass and leaf area index (LAI) with observations of stand structure for each site. While these comparisons are only semi-quantitative since we do not have direct measurements of NPP or LAI trajectories through time, they provide an indication of whether the model can accurately represent spatial differences in site productivity prior to the drought and the relative likelihood of surviving the drought.

Once we have developed our modeling approach, we then used RHESSys-NSC to answer two questions: 1) what is the relationship between estimates of minimum NSC during drought and the fraction of gross primary production (GPP) allocated to NSC; and 2) how do differences in precipitation and temperature between stands along an 800 m elevation gradient influence the NSC pool and ultimately the likelihood of mortality? To answer the first question, we examine the sensitivity of model estimates of minimum NSC during drought to the two parameters that control the fraction of GPP allocated to NSC. We use this sensitivity analysis to identify parameters that produce patterns of minimum NSC that correlate with observations of mortality.

We perform this sensitivity analysis for two different model implementations: 1) a stand level implementation at each site that accounts for site specific variation in pre-drought biomass, climate, and soil characteristics; and 2) a tree level implementation in which we assume the same average biomass per unit area for all three sites. In the tree level implementation all sites have the same biomass and only climate inputs and soil parameters differ. The tree level implementation examines the impact of drought on a “tree” or a given biomass per unit area at each of the three sites. The stand level implementation, on the other hand, includes spatial differences in current biomass that result from historic, decadal effects of spatial variation in environmental drivers (e.g. the cooler-wetter climate at the high elevation site supports greater biomass prior to the drought). Considering both a stand (e.g. average effects of the drought given stand characteristics that evolved prior to the drought) and a tree level (e.g. effects for the same biomass per unit area at each of the different sites) implementation allows us to disentangle the effect of pre-drought biomass from spatial differences in the severity of drought as a result of gradients in precipitation, temperature and differences in soils. Our second question then examines the physical drivers that give rise to spatial differences in forest mortality risk. To answer the second question, we examine how model predictions of NSC, productivity, and mortality vary between high and low elevation sites for stand level implementations given different temperature and precipitation drivers.

### Model Implementation

RHESSys is a process-based model of coupled hydrologic and ecosystem processes, spatially modeling fluxes both within and between model unit grid cells of user-defined size. RHESSys has successfully modeled both hydrologic and carbon cycling behavior such as evapotranspiration, streamflow, net ecosystem CO_2_ exchange, and NPP in mountainous regions in North America and Europe [25], [26], [27], [28], [29], [30].

RHESSys models the vertical flux of water between the atmosphere and canopy, and the litter and soil layers [31]. Modeled hydrologic processes include infiltration, vertical drainage between unsaturated and saturated stores, and lateral and vertical redistribution of shallow groundwater to deeper groundwater stores. Spatial patterns of radiation, and atmospheric drivers such as temperature, precipitation, humidity, and wind can be provided as inputs or estimated using empirical functions (i.e. [32]). For each model unit grid cell - energy, wind, and water are attenuated through the aboveground canopy as a function of LAI. RHESSys distinguishes between canopy interception of diffuse and direct radiation and sunlit and shaded leaves [33]. Snowmelt is estimated using a combination of an energy budget approach for radiation-driven melt and a temperature index-based approach for latent heat-driven melt processes. Transpiration from the canopy and evaporation of intercepted water and soil and litter moisture are computed using the Penman–Monteith equation [34], where stomatal conductance is computed using species-specific multiplicative models [35] of radiation, vapor pressure deficit, rooting zone soil moisture, CO_2_, and air temperature. GPP is computed using the Farquhar equation [36]. Respiration is computed as a function of temperature and plant component nitrogen content [37], modified to account for adaptation to ambient temperature during growth [38]. Carbon and nutrient cycling sub-models are similar to those used in BIOME BGC [18] and Century N-Gas [39], respectively. RHESSys determines the fraction of net photosynthesis (gross photosynthesis minus maintenance and growth respiration of foliar, woody, and fine root biomass) allocated to leaves and stems based on stand age following Dickinson [40]. In this approach, the proportion of net photosynthesis allocated to leaves declines as LAI increases. Allocation to fine roots follows allocation to leaves and the remainder of net photosynthesis is allocated to stems. Leaf, fine-root, and branch turnover occur as a fixed proportion of the associated biomass components. Turnover rates and allocation parameters for ponderosa pine were taken from the RHESSys parameter database.

**Figure 1:**
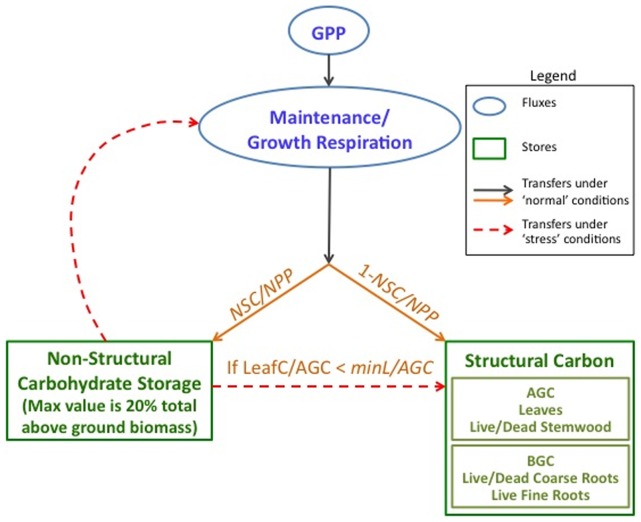
RHESSys submodel of allocation to NSC (non structural carbohydrate), with associated parameters. AGC is above ground plant structural carbon and BGC is below ground plant structural carbon. GPP is gross primary productivity. *NSC/NPP* is the fraction of annual NPP allocated to non-structural carbohydrate storage. *MinL/AGC* is defined as a threshold minimum for the ratio of leaf carbon (LeafC) to above ground carbon and determines when NSC will be depleted to maintain AGC.

We extended the current carbon cycling sub-model within RHESSys to explicitly model a within-tree NSC pool (Figure 1). This NSC pool maintains the net balance across years and includes two new parameters to control the non-structural carbohydrate allocation and consumption. *NSC/NPP* is the fraction of annual NPP allocated to non-structural carbohydrate storage until total NSC reaches a threshold percentage of plant structural carbon (20% of total plant biomass). Our 20% of total plant biomass threshold is based on field measurements as well as plant structural considerations [41], [42], [43], [44], [45]. An additional scheme was required for NSC allocation to respiration versus growth, particularly during periods of low GPP. Our approach was to assume trees utilize NSC reserves preferentially to maintain respiration, and once respiration requirements are met, the remaining NSC is used to replace fine roots and leaf turnover given that excessive reduction in these compartments during drought accelerates future mortality. To account for this use of NSC to replace leaf and fine root turnover, we examined a range of values of *MinL/AGC,* or the threshold minimum ratio of leaf carbon stores to total above ground carbon. If the leaf to above ground carbon falls below the threshold value defined by the *MinL/AGC* parameter, the tree will access NSC reserves after maintenance respiration requirements are met, to restore leaf biomass. Limited data on the allocation of NSC to fine roots versus foliage forced us to assume that fine root biomass is assumed to follow leaf biomass - such that allocation to leaves from NSC reserves also triggers allocation to fine roots. We are exploring additional flexibility in leaf/fine root allocation ratios in response to stress via subsequent work. We also examine model sensitivity to values of the *MinL/AGC* parameter ranging from 0 to 25%. The 25% upper limit for *MinL/AGC* is based on field measurements of leaf:aboveground biomass ratio for mature ponderosa pine stands [46]. We refer to this model as RHESSys-NSC throughout the remainder of the paper.

We track the time series of model estimates of NSC reserves and argue that mortality is increasingly likely to occur in conditions where our estimated NSC reserves approach zero. We use NSC here as an indicator of mortality risk and argue that low NSC reserve estimates in our carbon cycling model will correlate with a number of more complex physiological mechanisms that have been linked to drought related mortality including carbohydrate starvation, declines in the plant’s ability recover from cavitation and hydraulic failure, and reduction in allocation to defense mechanisms. Ultimately, we need to define a threshold NSC reserve, below which the likelihood of mortality increases. While the critical values of NSC below which mortality likelihood begins to increase remains uncertain, we assume that values below 0.5% of total above ground biomass are both physiologically realistic and will only occur under stress conditions that substantially reduce net accumulation of assimilates over time. For this paper we do not produce quantitative likelihood estimates for mortality, but rather use a fuzzy classification such that for stands with NSC below the 0.5% threshold the risk of mortality is high, for those near the 0.5% threshold the risk is moderate, and for those with NSC above the 0.5% threshold the likelihood of stand level mortality events is low. To test this use of NSC as an indicator of the relative likelihood of mortality, we compute NSC estimates for stands along an elevational gradient and compare the NSC estimates for sites that survived the 2000’s drought with those in which 100% mortality occurred.

### RHESSys Implementation for the New Mexico Study Watershed

We tested the new RHESSYs-NSC using ponderosa pine stands in the Jemez Mountains of northern New Mexico. This is a semi-arid continental climate region characterized by a bimodal precipitation pattern of summer and winter peaks, with strong elevational gradients of temperature and precipitation. We focus on three sites located across an 800 m elevation gradient within the Frijoles watershed of Bandelier National Monument, in the Jemez Mountains of northern New Mexico (Table 1 provides additional information on site characteristics. Sites have similar slope and aspect). The low elevation site is a relatively open stand of ponderosa pine mixed with one-seed juniper (*Juniperus monosperma*) and piñon pine (*Pinus edulis*) (Table 2 provides stand basal area (BA)). The mid-elevation site is closed canopy, even-aged ponderosa pine, and the high-elevation site is a relatively dense canopy site of mixed ponderosa pine, Douglas-Fir (*Pseudotsuga menziesii*) and aspen (*Populus tremuloides*). Observed ponderosa pine basal area was similar for the upper and mid site and substantially smaller for the low elevation site (Table 2). Although ponderosa pine BA is similar for high and low elevation sites, overall forest stand BA is greater for the high elevation site due to the presence of Douglas-fir and aspen trees. All stands are mature with ages between 95 and 300 years. In the simulation we modeled the high site stand as homogeneous ponderosa pine.

**Table 1:** Site characteristics and soil parameters.

Site	Elev (m)	Precip scaler (dim*)	Soil maximum saturated hydraulic conductivity (m/day)	Soil decay of saturated hydraulic conductivity with depth (m)	Soil Ψ air entry (m)	Soil pore size index (dim)
High	2767	1.27	764	8.75	0.159	0.16
Mid	2308	1.03	1500	4	0.175	0.168
Low	2002	0.85	3667	0.38	0.125	0.139

* dim  =  dimensionless, Precip scalar is the multiplier on input daily precipitation data to adjust for cross site differences in rainfall inputs.

**Table 2:** Modeled and observed stand characteristics prior to the drought.

Site	LAI after 100 year spinup (averaged over 20 years) (m^2^/m^2^)	Pearson Correlation Coef (mean across all parameters) NPP vs. BAI	Observed Mean BAI (prior to 2000) (cm^2^/yr)	Observed Stand Basal Area (BA) (m^2^/ha)
High	2.4	0.45 (0.37–0.53)	35.3	29
Mid	2.0	0.75 (0.71–0.82)	19.6	32
Low	1.6	0.73 (0.68–0.83)	15.1	8.8

LAI is modeled leaf area index. BAI is measured annual basal area increment. NPP is model estimate of net primary productivity.

At each site, dendrometer bands have monitored weekly stem growth for ten ponderosa pine trees per site since 1991, from which basal area increment (BAI) was calculated [20]. In late 2002, after three years of drought, all monitored ponderosa pine trees at the low elevation site died. In contrast, while mid and high elevation sites showed BAI declines during the drought, these pines did not experience significant mortality. BAI, mortality and survival are the key observed variables we used to test the RHESSys-NSC model performance. While methods are available to translate BAI into stem-wood growth for comparison to modeled stemwood accumulation, we argue that uncertainties in allometric parameters are high, since no on-site allometrics are available, ultimately reducing the information content of such a comparison. Correlation between model estimates of NPP and BAI however, demonstrates whether or not the model generally captures site and inter-annual differences in productivity.

Daily maximum and minimum air temperature and precipitation inputs to RHESSys are derived from the nearby Los Alamos meteorology station (elevation 2262m). To estimate precipitation inputs for each of the sites across the elevation gradient, we apply an elevation-based precipitation adjustment (Precip-scalar in Table 1) to meteorology station records. We derive this precipitation adjustment from historic records available at two additional local stations located at 2896 m and 3231 m (Quemazon snotel and Bandelier fire tower stations, respectively). Records for these sites are shorter than for the primary station so they could not be used as input time series. Final precipitation scalars used are summarized in Table 1. Note that while a standard environmental lapse rate for temperature (6.5 °C/km) is commonly used in hydrologic modeling, recent studies show that in the mountainous West, lapse rates are often substantially less than environmental lapse rates and typically differ for maximum and minimum temperatures [47]. To account for this, temperature inputs are also adjusted for elevation using relationships among the three meteorology station records. Computed lapse rates for this site are –5.7°C per km and –5.3°C per km for maximum and minimum temperatures, respectively.

Soils at the upper site are Pachic Argiustolls (Lucito loam on 1–8% slopes and Mapache gravelly loam on 10–25% slopes), at the mid site the soils are mixed Vitrandic Haplustolls and Typic Paleustalfs (Adornado-Letrado complex on 5–20% slopes) and typic Haplustalfs (Jemez sandy loam on 2–8% slopes), and at the low site the soils are Vitrandic Haplustolls (Adornado very paragravelly ashy loamy coarse sand on 8–15% slopes). RHESSys soil parameters for each site (Table1) were derived from recent soil survey data [48]. Soils at the upper site have a slower drainage rate and greater water holding capacity relative to the lower site. The mid site soils have the highest water holding capacity but have drainage rates intermediate between upper and lower sites.

To initialize forest structural carbon (in leaves, roots and stems), we ran a 100-year spinup simulation. Meteorology data for the spinup period is synthesized by repeating the available 50-year (water year 1950 to 2000) Los Alamos meteorologic station record, using adjustments for elevation as discussed above. Modeling unit resolution for these simulations is a 30 m grid cell. Thus, at each site stand carbon cycling dynamics are modeled as per unit area carbon store and fluxes averaged over a 30 m grid cell with mean elevation, climate and soil characteristics found at each of the three sites. Use of the historic spinup allows the model to account for spatial differences in stand characteristics at each site, assuming similar ages since disturbance. In these stand-level simulations we include a 5% background whole tree (stem, leaf, stem and coarse root) turnover [49]. Subsequent simulations based on this spinup include the effect of a-priori differences in stand biomass at each site in response to climate variation and drought. This means, for example, that the upper elevation stand may have greater total biomass, and thus typically utilize more water when aggregated over a 30 m modeling unit. To assess performance of the carbon-cycling model across the elevational gradient, we compared predictions of above ground biomass (total leaf and stem) for the three sites to stand measurements of BA and LAI. We also compared modeled NPP and observed BAI for all sites to assess model ability to capture response to inter-annual variation in climate conditions.

### Sensitivity analyses

Given the relative lack of empirical information regarding NSC dynamics during drought and prior to mortality, we performed sensitivity analyses of model growth predictions (NPP, change in LAI) and NSC to assess how much the different assumptions of NSC dynamics influenced predictions. Specifically, we varied *NSC/NPP* and *MinL/AGC* parameters to examine the sensitivity of model predictions of NSC and NPP. We began simulations in 1985 to account for any transient dynamics associated with shifting to a specific NSC parameter set. We then examine the model predictions for water years 1992 to 2007. In particular we compare the minimum NSC obtained for 1992–2007 to indicate likelihood of carbon starvation across the elevational gradient and across different values for storage parameters.

We perform sensitivity analysis for two different scenarios; one to estimate stand-level responses using spatially variable structural carbon (based on model spinup of leaves, stems and roots as described above) and a second to estimate tree-specific responses where initial biomass in leaf, stem and root compartments are set to be uniform across sites. We do this to disentangle differences in drought stress response that include the effect of apriori difference in biomass and biomass partitioning into leaves, stems and roots (stand-level response), and those due only to differences in site meteorology and soil conditions (tree-specific response). For tree-specific scenarios we use biomass (leaves, stems, and roots) components from the spinup for the mid elevation site and normalized the results by stand biomass. For tree-specific scenarios we assume no turnover of stemwood to reflect tree-based rather than stand-based estimates.

We used the results from the sensitivity analyses to select reasonable values of *NSC/NPP* and *MinL/AGC* parameters. We choose parameters that: 1) are consistent with cross-site and inter-annual patterns of observed BAI; 2) result in minimum values of NSC that are less than 0.5% of biomass for the low elevation site during drought; and 3) are physiologically and ecologically realistic. While the critical values of NSC below which mortality is likely to occur are uncertain, we assume that values below 0.5% indicate a high likelihood of stress related mortality. We then use this parameter set to run additional simulations, where we vary precipitation and temperature inputs and soil parameters to explore interactions among these controls on carbon cycling dynamics along the elevational gradient in more detail. For simplicity, we focus only on comparison between the high and low elevation sites. To compare the relative importance of temperature and precipitation dynamics, we contrast productivity estimates for simulations using spatially uniform structural carbon for the 1992 through 2007 period using a) the original meteorological dataset, b) simulations where all sites receive high site precipitation, c) simulations where all sites receive low site precipitation, d) simulations where all precipitation is assumed to fall as rain and temperature is unchanged, e) simulations where all sites use high site temperature, and f) simulations where all sites use low site temperature.

## Results

### Assessment of RHESSys biomass and productivity predictions across three sites

**Figure 2:**
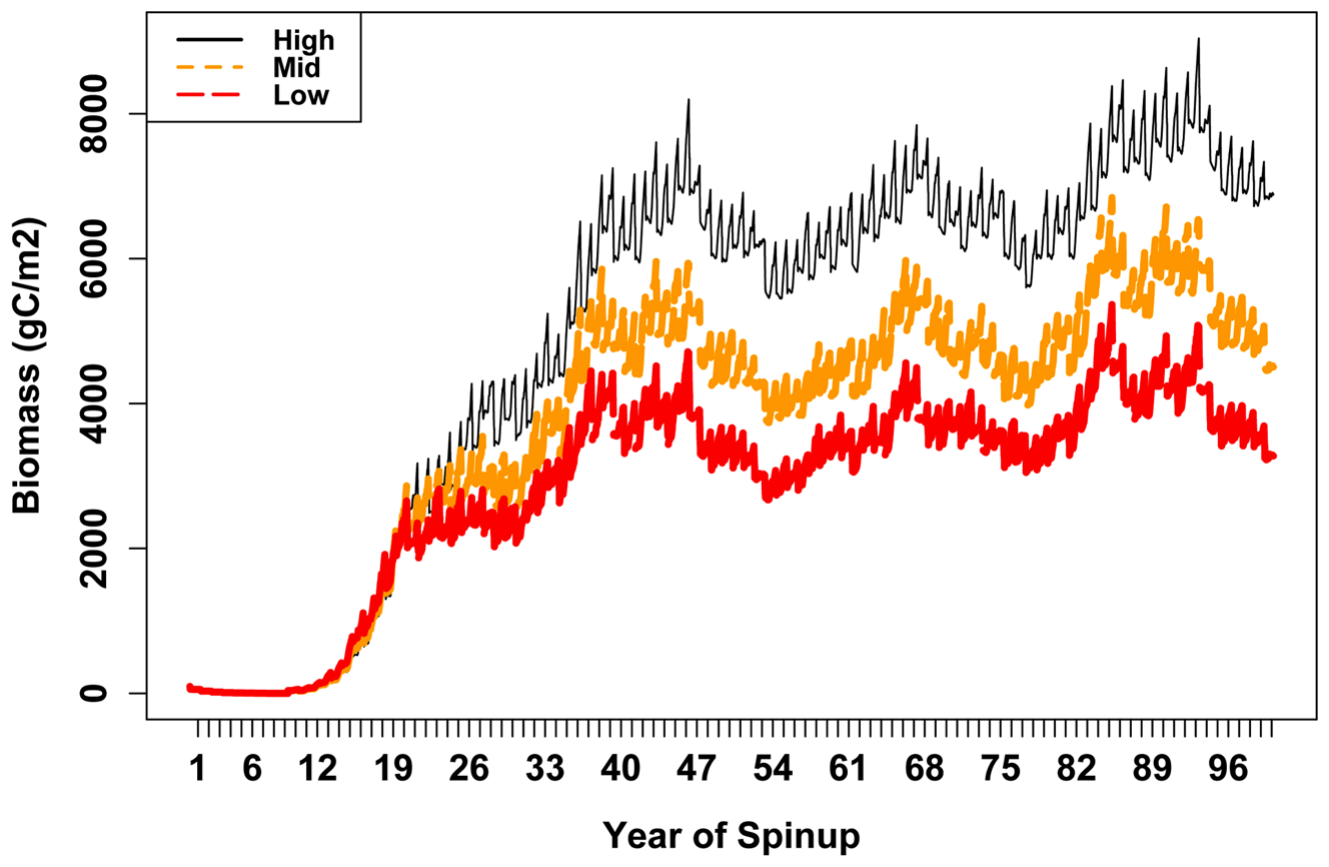
RHESSys estimates of total stand biomass for spinup period of 100 years.

**Figure 3:**
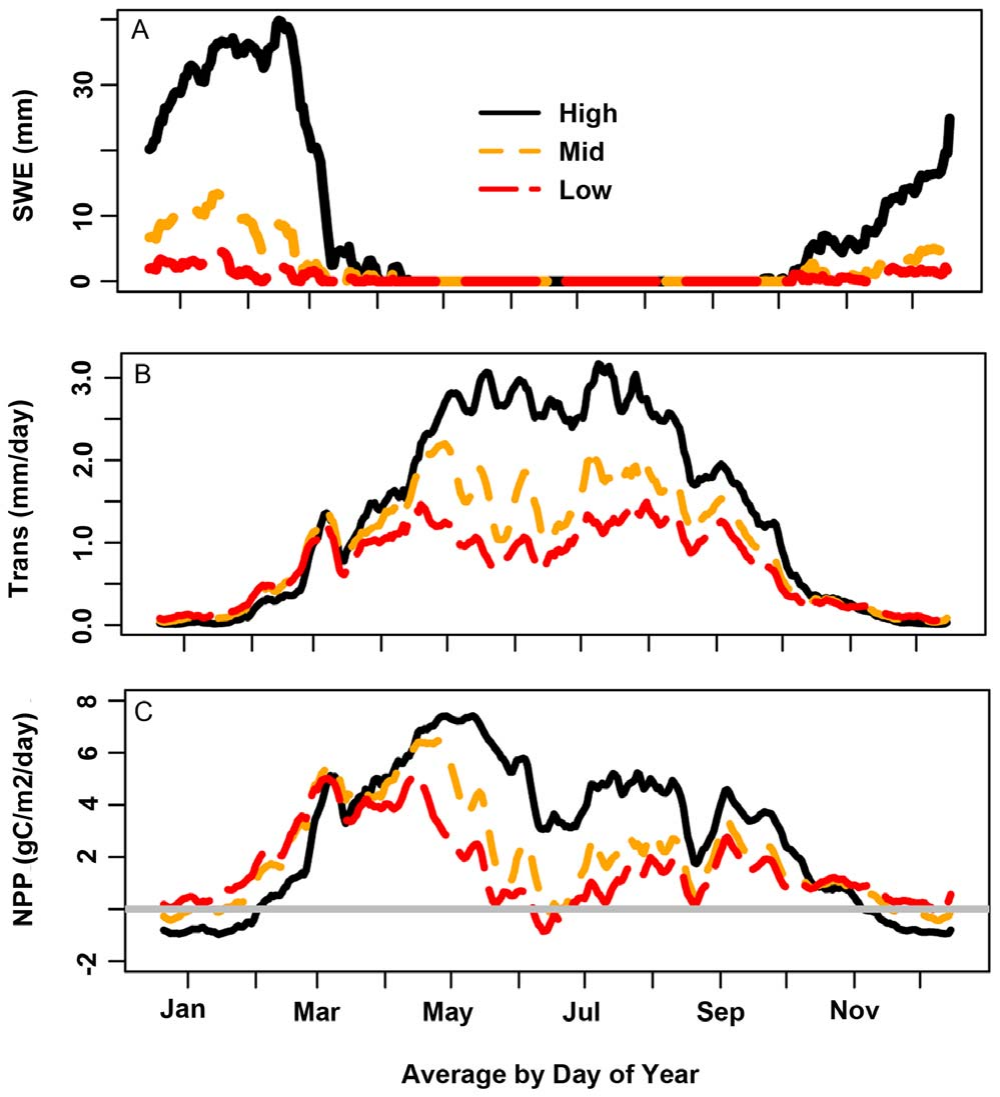
RHESSys estimates ecohydrologic fluxes for simulation period Graphs show a) snow water equivalent (per area of 30 m grid cell) for high, mid and low elevation site for water year 1992–2007 simulation period b) daily transpiration per grid cell, averaged by day of water year for pre-drought (1985–2000) period and c) daily net primary productivity (NPP) per grid cell, averaged by water year for pre-drought (1985–1999) period.

Model spinup leads to relatively stable biomass after approximately 50 years (Figure 2). The spinup estimates of LAI, biomass, and NPP are consistent with observed biomass and BAI across the elevation transect of the study site (Tables 2, and Figures 2, 3bc). The accuracy of the spinup simulations, along with the convergence of above and below ground biomass estimates to a relatively stable value prior to the drought, suggests that simulations are not likely to be sensitive to initial conditions or the age assumed at the beginning of the sensitivity analysis. The model estimates of biomass, NPP and LAI show increasing values with elevation that are consistent with observed patterns (Table 2, Figure 3c).

Model estimates show clear elevational differences in estimates of snow accumulation and melt, transpiration, and NPP (Figure 3). The high elevation site receives considerably more of its precipitation as snow relative to mid and low elevation sites (Table 1, Figure 3a). There is higher spring transpiration and NPP in the low elevation site, but lower summer transpiration and NPP - reflecting elevation differences in timing and magnitude of soil water and evaporative demand (Figure 3bc).

**Figure 4:**
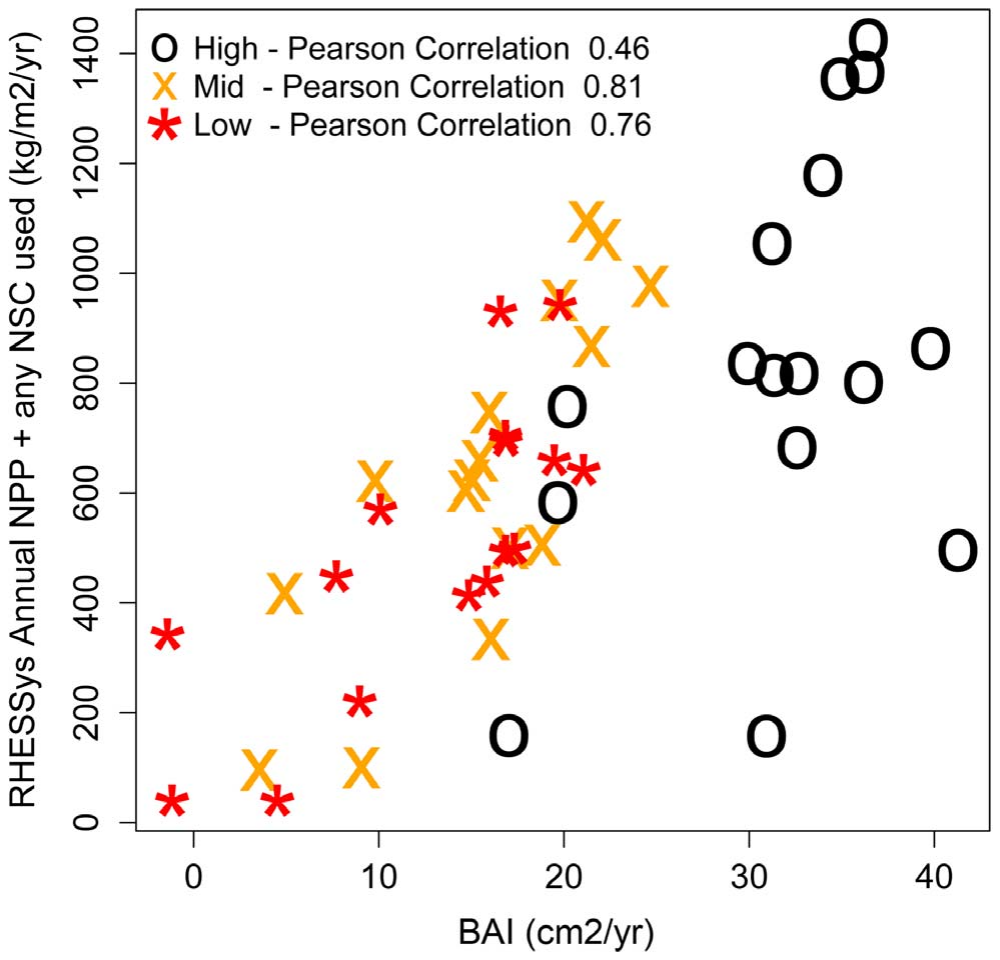
Comparison between observed basal area increment (BAI) and RHESSys estimates of annual net primary productivity (NPP). Points compare observed BAI and model NPP estimates for each year for the 1992–2007 period. Note in the NPP estimate we include the addition of any NSC that was removed from long term storage in a given year and used for growth. Here we report Pearson correlation coefficients using 0.3 and 0.05 for *NSC/NPP* and *MinL/AGC,* respectively. All correlation coefficients have p-value < 0.05. Table 2 provides mean and range of correlation coefficients for all NSC parameter values.

The relatively strong (>0.5) correlations between observed inter-annual BAI and modeled growth as NPP, plus any depletion of NSC pool for growth, suggest that major controls on inter-annual variation in productivity are captured (Table 2, Figure 4). These correlations remain above 0.4 regardless of assumptions of the parameters used for NSC (discussed below) and suggest that estimates of NPP are robust across this parameter uncertainty. In other words, uncertainty in NSC-related parameters does not preclude the use of the model to obtain realistic estimates of year-to-year variability in NPP for pre-drought years.

### Sensitivity Analyses: Stand level simulations with cross-site differences in biomass

**Figure 5:**
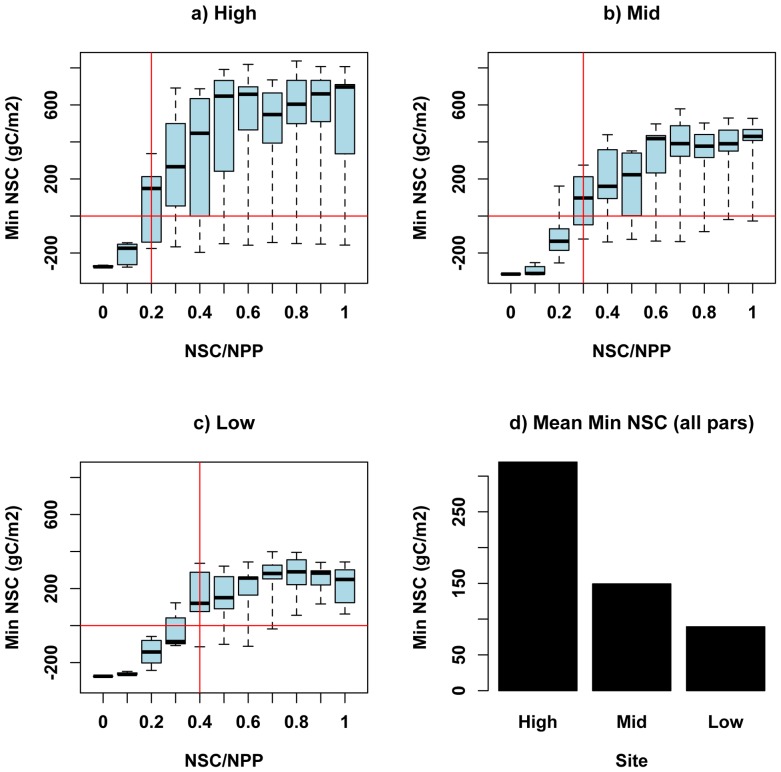
Estimates of minimum NSC over 1992–2007 simulation period for high, mid and low elevation stands. Graphs show variation in minimum NSC estimates across variation in parameters: *NSC/NPP* (x-axis) and *MinL/AGC* (box widths). Horizontal lines denote values of NSC below which mortality is likely. Vertical line denotes value of *NSC/NPP* parameter for which minimum NSC is greater than zero for most values (3^rd^ quartile) of *MinL/AGC*. This value of *NSC/NPP* is 0.28, 0.32, 0.38 for high, mid and low sites respectively. Bottom right graph shows average value of minimum NSC across all parameters for high, mid and low elevation sites. Results are based on simulations using carbon storage initialized through 100 yr spinup.

For stand-level simulations, minimum NSC over the simulation period (1992 to 2007) varies with NSC allocation parameters for all sites (Figure 5). Minimum NSC occurs during the early 2000’s drought for all sites and parameter sets. There is a tradeoff between allocation to maintain carbohydrate storage reserves, which reduces drought vulnerability, and allocating to growth, which increases photosynthetic capacity. Thus, lower values for the *MinL/AGC* parameter allow greater loss of LAI during drought but maintain greater reserves and ultimately reduce mortality risk associated with low NSC values. A value of 0 for *MinL/AGC* reflects the scenario where trees use non-structural carbohydrate reserves to maintain respiration costs only, and do not produce foliage or fine roots from these reserves. Greater allocation to NSC storage (the *NSC/NPP* parameter) lead to higher values for the minimum NSC parameter, although for all three sites there is a threshold value for *NSC/NPP* above which increases in *NSC/NPP* do not lead to higher values of minimum NSC (Figure 5). This upper threshold results from interactions with the minimum leaf area (the *MinL/AGC* parameter) requirement and maximum allowable NSC (20% of total biomass), which also limits allocation to NSC when reserves are high.

**Figure 6:**
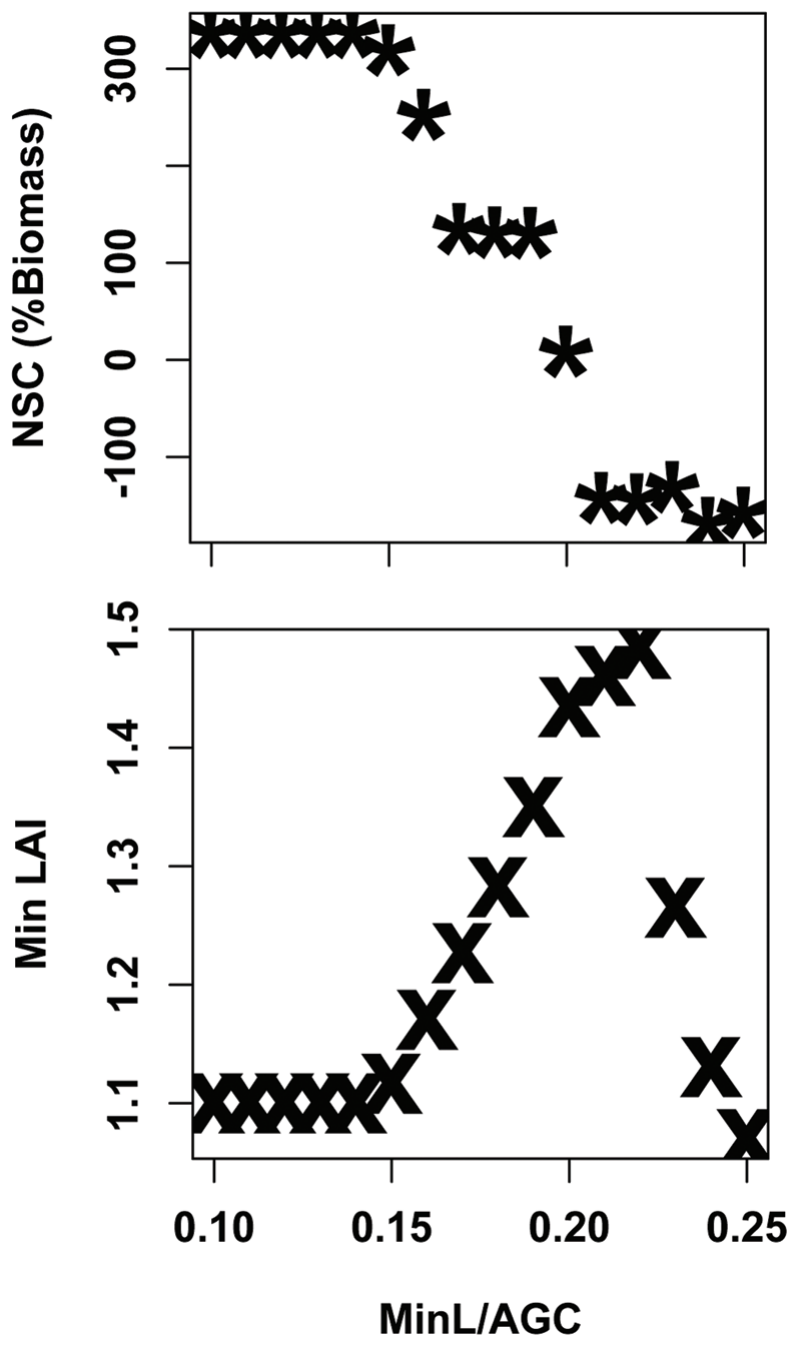
Estimates of a) minimum NSC and b) minimum LAI over 1992–2007 simulation period for high elevation stands. Graphs show variation in minimum NSC and LAI across variation in *MinL/AGC* with *NSC/NPP* set equal to 0.25.

Sensitivity of NSC to the *MinL/AGC* parameter varies with *NSC/NPP*. In general, lower values of *MinL/AGC* lead to higher NSC. The greatest sensitivity of minimum NSC estimates to *MinL/AGC* occurs at intermediate values of *NSC/NPP*. For lower *NSC/NPP* values, minimum NSC is strongly controlled by plant NPP, and differences due to the *MinL/AGC* parameter become negligible. At higher values of NSC/NPP, NSC becomes large enough to support a range of *MinL/AGC* requirements. For intermediate values of NSC/NPP, sensitivity in the *MinL/AGC* parameter reflects tradeoffs between maintaining leaf area and carbohydrate storage. Figure 6 demonstrates this trade-off for the upper elevation site. For values of *MinL/AGC* above 0.15, the minimum LAI over the full 1992–2007 period increases until a threshold is reached. Above this threshold, NSC allocation to leaves in good years ultimately leads to greater vulnerability to drought, and results in lower minimum LAI and minimum NSC. Higher, and probably physiologically unrealistic values of *MinL/AGC* (> 0.2), lead to mortality even in the high elevation site as the tree rapidly depletes NSC to maintain leaf area.

The minimum predicted NSC increases with elevation for all simulations except when *NSC/NPP* is set extremely low, in which case all sites achieve zero NSC. Thus, for most of our NSC allocation assumptions, the model estimates greater vulnerability to drought stress for the lower elevation sites (Figure 5). This spatial trend in minimum NSC is maintained after normalizing minimum NSC by total biomass. Here we note that if we assume a critical value for NSC of 0.5% of biomass as indicative of mortality, values for the *NSC/NPP* parameter between 0.24 and 0.36 correctly suggest mortality at the low elevation, but not mid to high elevation sites. This suggests that parameter values within this range provide the most realistic assumptions about NSC storage dynamics. The strong correlations of modeled NPP and observed BAI (Table 2) for most parameter sets suggests simulation of growth is robust across parameter uncertainty. Thus, while most NSC parameter sets correctly captured inter-annual and spatial variation in growth, only *NSC/NPP* values near 0.3 captured differences in the likelihood of drought related mortality.

### Sensitivity Analyses: Tree level simulations with uniform biomass for all three sites

**Figure 7:**
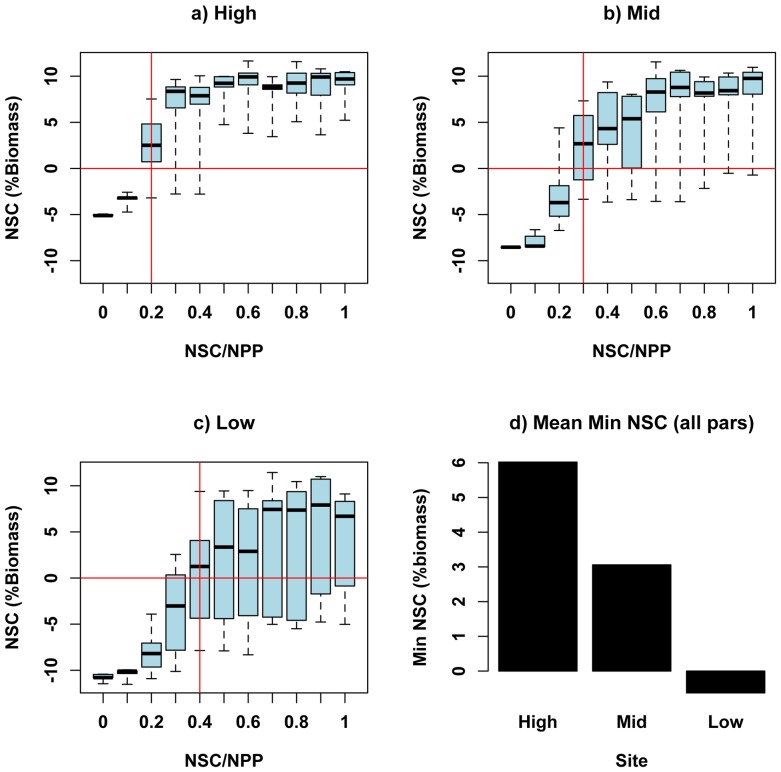
Estimates of minimum NSC as a percentage of biomass over 1992–2007 simulation period. Estimates of minimum NSC are shown for a tree of similar size for high, mid and low elevation sites. Horizontal lines denote values of NSC below which mortality is likely. Vertical lines denote the value of the *NSC/NPP* parameter for which minimum NSC is greater than zero for most values (3^rd^ quartile) of *MinL/AGC*. Graphs show variation in minimum NSC across variation in parameters: *NSC/NPP* (x-axis) and *MinL/AGC* (box widths). Bottom right graph shows average value of minimum NSC across all parameters for high, mid and low elevation site. Initial structural carbon values are the same for all three sites.

Results from the sensitivity analysis for tree-level simulations, where initial above and below ground structural carbon are spatially uniform, show similar patterns to those obtained for stand level simulations when structural carbon is varied across sites (Figure 7). As with spatially variable biomass simulations, estimates of minimum NSC stores are consistently less for the low elevation site across all parameter sets, with greater minimum NSC for mid rather than high elevation sites (Figure 7). Again, the threshold value of *NSC/NPP* above which NSC does not fall below our critical mortality threshold (0.5%), is lower for mid and high elevation sites, suggesting these stands can survive with relatively less allocation to NSC than the low site (Figure 7). The greatest cross-site differences in the attribution of mortality (storage < 0.5%) are found for *NSC/NPP* values of 0.2 to 0.4. For these tree-level simulations we used an initial biomass consistent with the mid-elevation site. In general, greater biomass (of individual trees or total stand) would be expected to increase respiration costs and water use and potentially increase the rate of NSC depletion during periods of drought and low GPP. Consistent with this, stand level simulations (Figure 5) for the high elevation site, where initial structural carbon values are greater, do show slightly lower minimum NSC relative to tree level simulations (Figure 7) for the same *NSC/NPP* parameter value. This difference, however, is relatively small and suggests that site condition differences (soil, meteorology) rather than pre-drought differences in stand structure, are the dominant controls on cross-site patterns of NSC.

**Figure 8:**
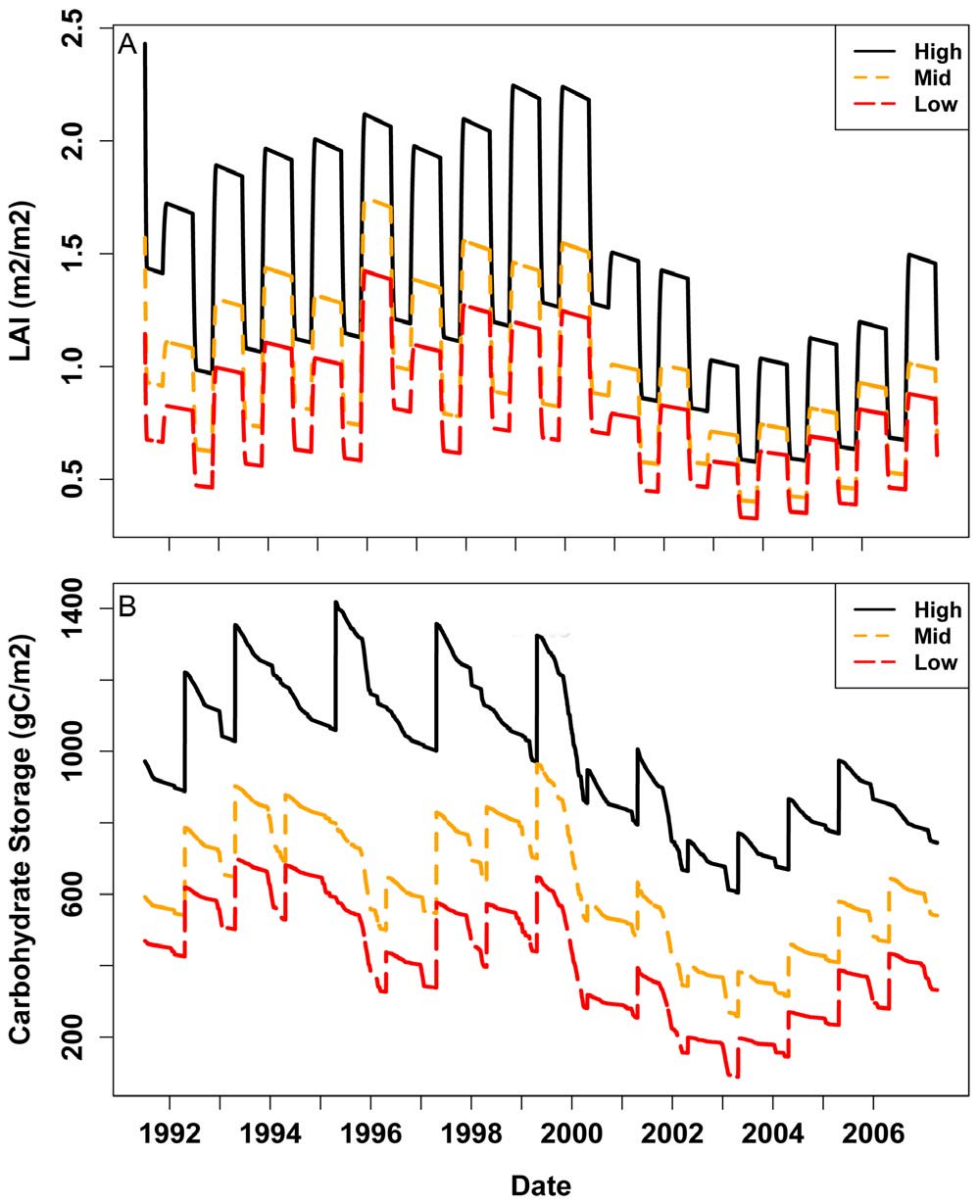
Trajectory of a) LAI and b) Non Structural Carbohydrate Storage. Trajectories of LAI and NSC are shown for high, mid and low elevation sites for 1992–2007 period. *NSC/NPP* and *MinL/AGC* are set to 0.3 and 0.05 respectively. We note that for the low elevation site, we predict that mortality would occur during the drought based on low NSC values. Based on this we could trigger a reduction of LAI to zero. We show predicted LAI without this mortality trigger to illustrate the response of the carbon cycling model and to point out that simply tracking needle loss would not be sufficient to capture the mortality event.

In summary, our sensitivity analysis suggests values for the *NSC/NPP* parameter between 0.25 and 0.35 correctly predict the timing and magnitude of high mortality likelihood at the low elevation site while still allowing growth variation in the mid and high elevation sites to track observed values. For the *MinL/AGC* parameter, a wide range of values give reasonable NSC and growth predictions when combined with NSC/NPP parameter values in the 0.25 to 0.35 range. Field studies, however, suggest that observed minimum values for the ratio of leaf to above ground biomass are close to 0.05 (5%) [46], thus we set *MinL/AGC* to this value. While we do not have measurements of LAI and NSC throughout the drought, we argue that the trajectory of LAI and NSC using *NSC/NPP* of 0.25 and *MinL/AGC* of 0.05 (Figure 8ab) are consistent with observations that the mortality occurred only at the low elevation site and that productivity, prior to the drought, increased with elevation (as indicated by increasing mean measured BAI (Table 2)). Model estimates of NPP also show a good correlation between observed and modeled BAI (Table 2, Figure 4). Results confirm that our model and parameter sets are both reasonable from a physiological perspective and produce simulations that correctly capture observed differences in the likelihood of drought stress mortality during the 2000’s drought.

### Impact of differences in precipitation and temperature across sites on NSC and mortality patterns

To examine climate controls on cross-site differences in growth and survival we run simulations using parameter values for *NSC/NPP* of 0.25 and *MinL/AGC* of 5%. Differences in simulation results between the high and low elevation sites reflect complex interactions between air temperature and precipitation on productivity and NSC (Table 3). Despite the expected shifts in NPP by applying low elevation precipitation to the high site and vice versa, we observed that these changes in precipitation do not account fully for differences in NPP. Using air temperature from the low elevation site for the high elevation site only slightly decreases NPP relative to the baseline scenario. We note that temperature influences the model estimates of productivity directly through impacts on respiration and photosynthesis sub-models as well as indirectly though impacts on snow accumulation and melt and evaporative forcing. The combination of greater water holding capacity in the soils and greater precipitation at the high elevation site supports greater productivity at higher temperatures. For the low elevation site, using cooler temperatures leads to relatively small declines in productivity. This site is more clearly water limited, even when temperatures from the high elevation site are used. A shift from snow to rain at the high elevation site only slightly reduces NPP.

**Table 3:** Change in estimated NPP with climate forcing conditions.

NPP (gC/m2/yr) Averaged for Water Years 1992–2004				
		Exchange of forcing data between High and Low site		
Site	Baseline	Precipitation fromother site	Temperature fromother site	Precipitation andTemperature from other site
High	2.00	1.30	2.10	1.27
Low	1.01	1.80	1.05	1.82

**Figure 9:**
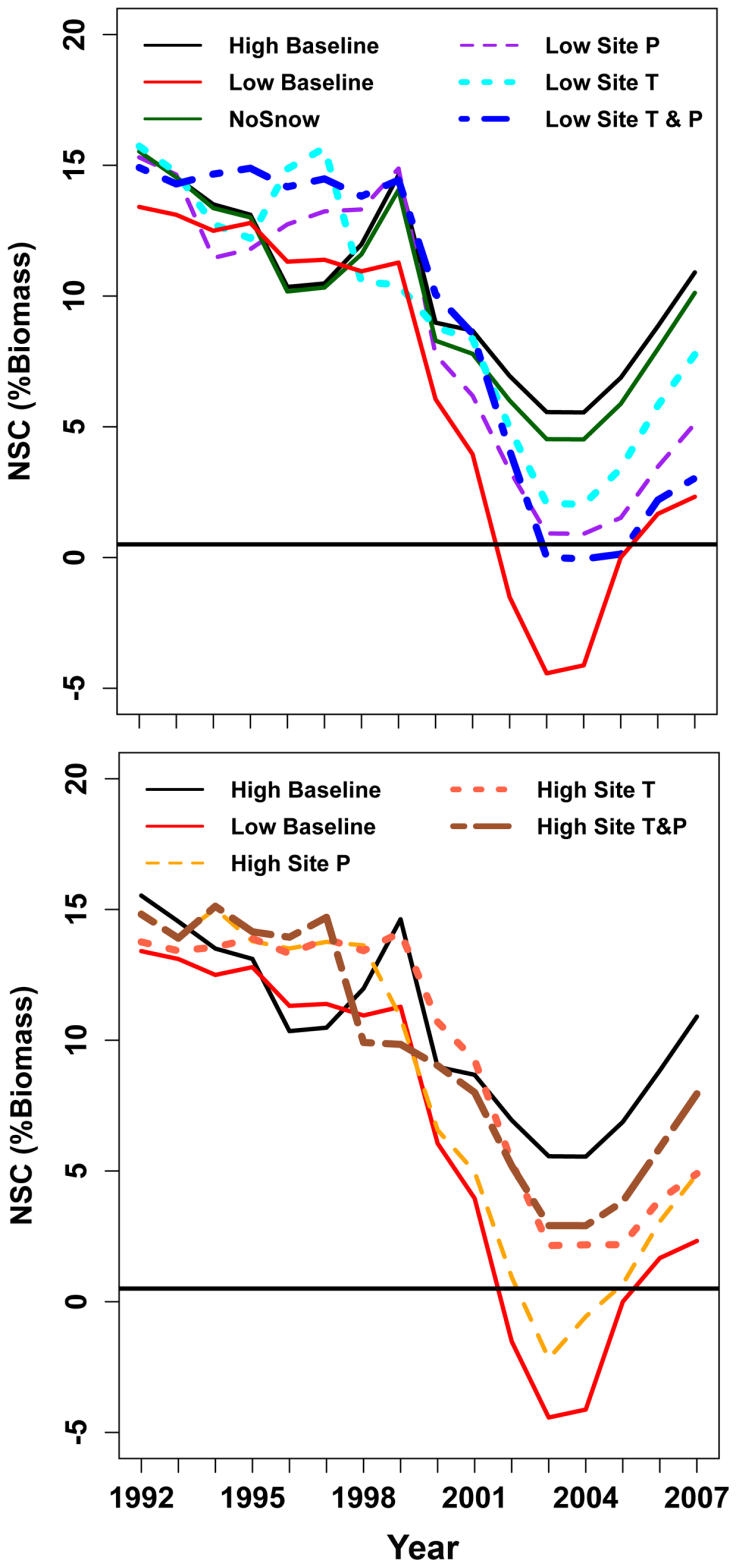
Trajectories of NSC through the 1992–2004 simulation period. Graphs of NSC trajectories are shown for both the pre-drought and drought years. *NSC/NPP* and *MinL/AGC* are set to 0.3 and 0.05 respectively and structural carbon estimates are initialized to be uniform across all 3 sites. Results are shown for the high site for 5 scenarios: baseline, all precipitation falls as rain (no snow), precipitation from low elevation site, temperature from low elevation site and both temperature and precipitation from the low elevation site. For the low elevation site 4 scenarios are shown: baseline, precipitation from high elevation site, temperature from high elevation site and both temperature and precipitation from the high elevation site.

NSC and associated estimates of mortality risk show a different response pattern to temperature changes than the productivity patterns (Figure 9). NSC drops close to the mortality threshold for the high elevation site if either low elevation precipitation or low elevation temperature is used (dotted blue and purple lines in Figure 9a). The impact of snow versus rain is relatively small but can be seen early in the 2000 drought for the high elevation site where there is a steeper decline in NSC if all precipitation is assumed to fall as rain. For the low elevation site (Figure 9b), using high site precipitation delays, but does not prevent, mortality in the 2000 drought years; using the high elevation temperature, however, does. For the low elevation site, if both high precipitation and temperature are used, NSC remains above the mortality threshold; although it is still below high site values reflecting the impact of lower soil water holding capacity at the low elevation site.

**Figure 10:**
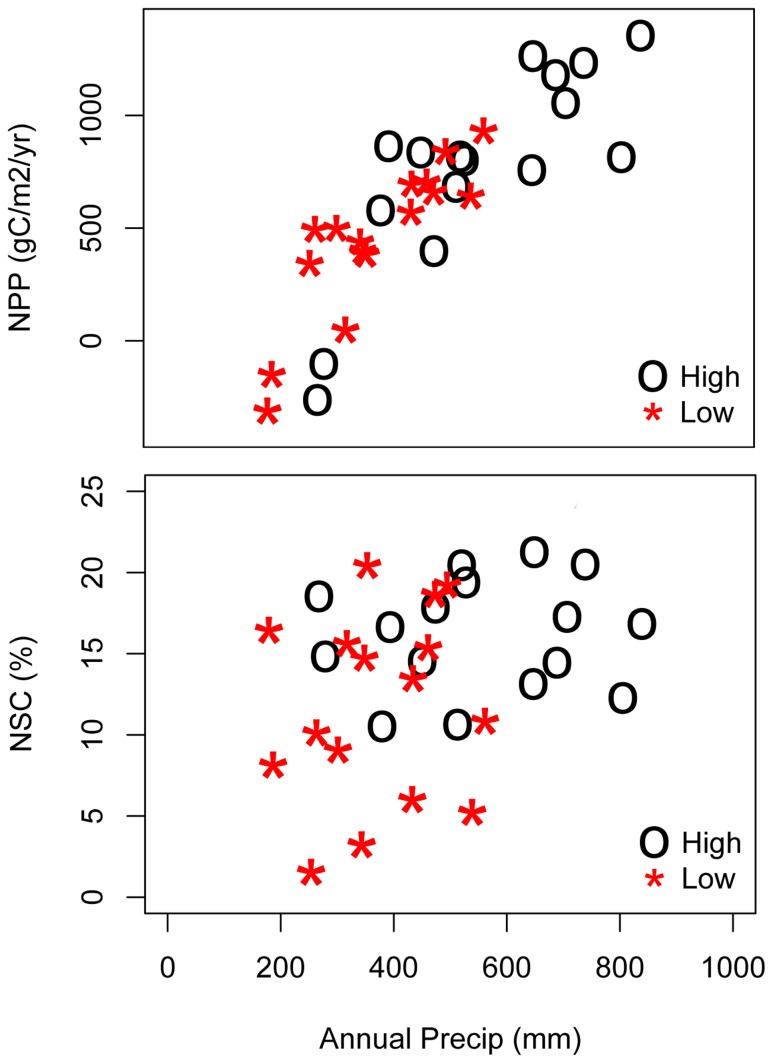
NPP and NSC against precipitation for high and low elevation sites over 1992-2007 simulation period.

This distinction between NSC and NPP behavior can be further explored by looking at model estimates of the relationship between NSC or NPP and annual precipitation (Figure 10). For NPP, upper and lower sites show a similar near linear relationship with annual precipitation until a threshold precipitation is reached (this threshold only occurs for the high elevation site). NSC integrates productivity over several years and results in a less direct relationship with precipitation.

## Discussion

Our objective was to determine if a simple NSC model embedded within a coupled carbon-hydrologic model (RHESSys-NSC) can capture observed mortality during drought, and if successful, what does this tell us about how climate controls influence mortality along a relatively fine-scale (800 m) elevation gradient? []Our results demonstrate a successful implementation of a NSC model into RHESSys such that the model accurately predicts both inter-annual and spatial variation in productivity and likelihood of mortality during drought. Model estimates of GPP, respiration, and biomass components are also similar to observations for ponderosa pine in the Western US [50], [51]. Minimum NSC during drought showed a greater sensitivity to temperature differences than NPP. Notably, the model scenario in which the warmer low elevation temperatures drive the high elevation site showed only a slight decrease in NPP, but NSC would have fallen close to the mortality threshold during the drought. This has implications for predicting forest mortality risk in a changing climate where future droughts are likely to occur under warmer temperatures [52], [53], [54], [49].

Including a *NSC/NPP* ratio of 0.25 resulted in accurate simulations of both growth (based on correlations above 0.4 between observed BAI and modeled NPP) and low elevation site mortality across the 800 m elevational transect (Figure 8). We emphasize that resulting values for *NSC/NPP* also make physiological sense., There must be some allocation to NSC to maintain respiration so *NSC/NPP* must be greater than zero, however too much allocation to NSC (*NSC/NPP* or values much greater than 0.4) is less competitive given tradeoffs between using carbon to support storage versus growth. Thus, our sensitivity analysis shows that the best performing parameter sets are consistent with physically realistic NSC storage dynamics. Further, our results are consistent with a recent study in this watershed that concluded carbon assimilation was a major component of the growth and mortality response of ponderosa pine across these same three sites [20].

Our model results do not prove that carbon starvation (e.g. [20]) was the last step in mortality of these particular trees because hydraulic failure, attack by bark beetles (*Ips* species) or other factors may have triggered the final phases of mortality [55], [11], [56]. However, it is likely that declining NSC increases risks of non-recoverable cavitation [24] and promotes susceptibility to biotic mortality agents, as less carbon is available to allocate to defensive compounds [57], [58], [11]. Given this general support for energy-limitation as a key factor in mortality, we argue that this simple two-parameter NSC model provides a good approximation of mortality risk. Simplicity is important in the context of modeling for forest management and scenario development at watershed to landscape scales. While more detailed physiological-based models of mortality are emerging, they will be challenging to parameterize and apply over complex heterogeneous landscapes.

The modeled differences in NSC and NPP responses across the elevational transect with drought are not necessarily surprising, but illustrate the necessity of using a storage term (NSC in our case) to track conditions under which mortality is likely to occur. Previous models of tree scale mortality have similarly shown that short time scale estimates of deficits in net assimilation (photosynthesis minus respiration) occur frequently and do not correlate well with mortality [59]. We note that a large number of experimental datasets on conifers are now revealing that tree death is associated with severe depletions in NSC [23]. Recent experimental work, however, has also shown that the ability of forest to use available carbon resources or NSC can also be limited by temperature [60] and drought [61]. In these cases, NSC may not be fully depleted prior to death. In this study, we posit NSC as an index for more complex mechanisms. We argue that this general pattern of energy reserves and their depletion leading to mortality provides a first-order approximation to mortality risk, and is consistent with the hypothesis that NSC storage is either directly or indirectly related to mortality through its interdependence on hydraulics and biotic attack [11]. Even if the actual causes of mortality are physiological limitations not directly reflected by NSC depletion, our NSC index may serve as an indicator of the degree of stress. We argue that our relatively simple approach may be a useful one because the linkage between a carbon cycling model with a simple NSC storage term and a hydrology model can integrate the effect of environmental controls on both the longer term development of plant structure (including biomass, rooting depth, and photosynthetic capacity) and short term stress due to physical controls on moisture availability (e.g. vapor pressure deficit, soil water availability).

Future model developments that could benefit improved predictions, as well as enable investigation of physiological mechanisms, include temporally variable *NSC/NPP* (i.e. increased ratio during drought), as well as other proposed mechanisms of mortality, such as hydraulic failure, phloem transport failure, and overwhelming biotic attack [11], leaf shedding [62] and responses related to the incorporation of NSC into plant tissues [13], [61]. We note that a key uncertainty in the model is in the strategies used for allocation of net photosynthesis to different plant components including leaves and roots. While the assumptions used in this study (age-based reduction in allocation to leaves and fine-roots) result in biomass and NPP estimates that are consistent with observations, different allocation strategies may alter results and ultimately the sensitivity of model estimates to drought. Understanding plant allocation strategies remains an active area of research within the plant physiology literature [63]. Future work will examine the sensitivity of RHESSys-NSC drought responses to leaf and root allocation and senescence strategies.

Sensitivity analyses in this paper demonstrate the importance of within-watershed patterns of temperature and precipitation as controls on productivity and mortality. Thus, while representing plant physiological responses to water stress is important, combining these estimates with reasonable representation of local energy and moisture conditions also is a key characteristic of a useful model. Scenarios using our coupled model show that productivity/mortality risk may not always respond in parallel – such that increasing temperatures may increase both NPP and mortality risk – and that even within a watershed, temperature and moisture gradients combine in complex ways to shape responses.

An important component of the RHESSys modeling framework is the coupling between a hydrologic model that accounts for spatial difference in precipitation, snow accumulation and melt, and evapotranspiration/soil water controls on ecosystem carbon cycling and ultimately mortality. Modeling in this study focused on within watershed spatial patterns for three sites within a 20 km distance along an 800 m elevation gradient. We note that the ability to capture these within-watershed scale patterns is dependent on accurate resolution of temperature and precipitation patterns. Spatial differences in temperature and precipitation (as well as radiation and other atmospheric drivers) between these sites typically would not be resolved by global climate model output, and capturing high resolution (<50 km) spatial patterns in mountain environments remains a challenge for regional models. Techniques to improve downscaling of regional climate models remain an active area of research [64], [65], [66]. Our results demonstrate that accounting for these within-watershed spatial differences in microclimate clearly have important implications for estimates of watershed scale patterns of carbon cycling and water use.

Climate-induced tree mortality is emerging as an increasing concern for forest managers around the world [3], [49], [67], [68], and climate models project significant rises in global temperatures and increasing drought frequency and severity for many regions in this century, including much of the Southwest US [54], [69]. Models that link these climate projections with spatially explicit changes in forest productivity and tree mortality are needed to support adaptation planning and actions by forest managers [70], [71]. Results from this study demonstrate a successful application of a coupled ecohydrologic model in predicting forest behavior at scales relevant to park and local forest managers. Future work will use the model to estimate mortality risk for a broad range of climate and climate change scenarios for the Jemez Mountains region and elsewhere. Application of models like RHESSys that account for within-watershed spatial variation in interactions among climate, hydrology and carbon can be important tools in watershed-scale assessments directed at supporting climate change adaptation.
